# Supplementary material for: Complexome profiling of the Chlamydomonas psb28 mutant reveals TEF5 as an early PSII assembly factor
Source: Plant Cell. 2025 Mar 17;37(6):koaf055. doi: 10.1093/plcell/koaf055 (PMC12164590; doi:10.1093/plcell/koaf055)
Supplement: koaf055_Supplementary_Data [file koaf055_supplementary_data.zip › psb28_interactive.zip!/plots/intensity/qualityPlots/peptides/COP3_Cre14.g611300.t1.1.html]

### Cre14.g611300.t1.1

Trivial name: COP3  
SeqCoverage [median]: 11.30  
Mass: 77.37 kDa  
  
 

| Sequence | MissedCleavage | Mass [Da] | wtIntensity | muIntensity | Score |
| --- | --- | --- | --- | --- | --- |
| LKNELGE | 1 | 801.42 | 0.00 | 863.20 | 128.86 |
| SPTGLLPR | 0 | 839.49 | 0.00 | 1524.34 | 128.57 |
| VNVAGQEMEVETMVHEEDDETQKVPTAK | 1 | 3142.44 | 0.00 | 3100.71 | 86.79 |
| YANRDSFIIMR | 1 | 1384.69 | 0.00 | 837.11 | 78.65 |
|  |  |  | 0.0 | 6325.4 |  |
